# Ischemic Hepatitis as the Presenting Manifestation of Cardiac Amyloidosis

**DOI:** 10.1177/2324709614558064

**Published:** 2014-11-12

**Authors:** Chelsey A. Petz, Thomas Todoran, Don C. Rockey

**Affiliations:** 1 Riverside Methodist Hospital, Columbus, OH, USA; 2 Medical University of South Carolina, Charleston, SC, USA

**Keywords:** hypoxia, heart failure, infiltrative, ischemia, shock

## Abstract

An abrupt elevation in aminotransferases without clear etiology may be attributed to hypoxic hepatitis. Underlying cardiac dysfunction, an important clinical clue, is often overlooked as a cause of hypoxic hepatitis, and understanding the interdependence of the heart and liver is crucial in making this diagnosis. Causes of cardiac dysfunction may include any of many different diagnoses; infiltrative heart disease is a rare cause of cardiac dysfunction, with amyloidosis being the most common among this category of pathologies. More advanced imaging techniques have improved the ability to diagnose infiltrative heart disease, thus allowing quicker diagnosis of conditions such as amyloidosis.

## Introduction

An abrupt elevation in aminotransferases has a limited differential diagnosis. A thorough investigation into the cause of the elevation can lead to the discovery of other systemic processes. Recognizing the interdependence of different organs is crucial to understand disease processes and make the correct diagnosis.

### Case Presentation

A 78-year-old man presented with 1-day history of nausea and vomiting. His symptoms started suddenly the night prior to presentation. The nausea was constant, to the point where he could no longer tolerate oral intake. Vomiting occurred 5 to 6 times throughout the evening, but there were no further episodes as of the morning of presentation. He endorsed very minimal epigastric burning, which he described as a “gas pain,” but he had no other abdominal symptoms. His bowel movements were normal. The patient was discharged from the hospital 10 days prior; during that hospitalization, he had symptomatic bradycardia felt to be secondary to his β-blocker. His dosage was reduced, his bradycardia improved, and he was discharged home. Since discharge, he noted persistent exertional dyspnea and bilateral lower extremity edema.

The past medical history was remarkable for type 2 diabetes mellitus, hyperlipidemia, paroxysmal atrial fibrillation, nonischemic cardiomyopathy (ejection fraction of approximately 40%) with stage III diastolic dysfunction, and chronic kidney disease (baseline creatinine 2.2). He smoked 4 cigarettes daily for 25 years, but not the last 20 years. His medications included aspirin, simvastatin, furosemide, metoprolol tartrate, and glipizide.

On examination, his blood pressure was 136/71 mmHg, heart rate 77 bpm, respiratory rate 16, temperature 36.1°C, oxygen saturation 100% (room air), weight 82 kg, and no orthostasis. In general, he was not in acute distress. There were no skin lesions. Mucous membranes were moist. Lung fields were clear to auscultation bilaterally. Heart was regular without murmurs, rubs, or gallops. He had jugular venous distention 4 cm above the sternal angle. His abdomen was benign. He had no tenderness over his liver and no hepatomegaly was present. He had minimal bilateral lower extremity edema.

The patient’s white blood cell count was 15 200/mm^3^. The hemoglobin was 13 g/dL, and the platelet count was 97 000/mm^3^. The total bilirubin was 3.3 mg/dL, direct bilirubin 1.3 µg/dL, aspartate aminotransferase (AST) 4725 IU/L (normal value = 12-38 IU/L), alanine aminotransferase (ALT) 3240 IU/L (normal value = 10-45 IU/L), alkaline phosphatase 110 IU/L (normal value = 25-100 IU/L), total protein 6.6 g/dL, and albumin 3.7 g/dL. The international normalized ratio (INR) was 2.6. His sodium was 134 mmol/L, potassium 5.6 mmol/L, bicarbonate 21 mmol/L, blood urea nitrogen 54 mg/dL, and creatinine 3.5 mg/dL. His brain-natriuretic peptide was 1040 pg/mL (normal = <100 pg/mL), and troponin was 0.50 ng/mL (normal = <0.09 mg/mL). His lipase was 49.0 IU/L (normal = 10-50 IU/L).

An abdominal ultrasound revealed increased echogenicity of the liver parenchyma, patent hepatic vasculature, and no dilated intrahepatic or extrahepatic ducts. The acetaminophen level was zero and the urine drug screen negative. Hepatitis A to C serologies were negative, and antinuclear antibody, antimitochondrial antibody, F-actin antibody, and liver-kidney microsome-1 antibody titers were all normal. Further review of his past records indicated that his liver tests were AST 47 IU/L and ALT 53 IU/L during his hospitalization 10 days ago.

**Figure 1.**
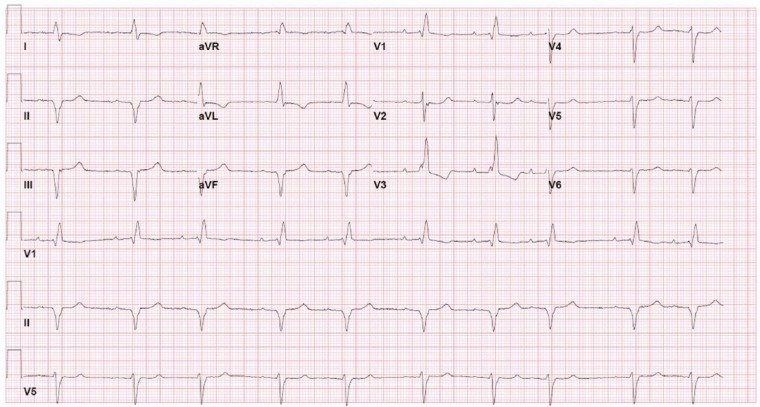
Electrocardiogram. This electrocardiogram reveals a number of conduction defects including a right bundle branch block, left anterior fasicular block, and a first-degree atrioventricular block. It also shows inferior Q waves and T wave inversions in lead I, aVL, and V3.

The patient’s electrocardiogram (ECG; Figure 1) revealed multiple conduction defects including a right bundle branch block, left anterior fascicular block, and a first-degree atrioventricular block. He also had inferior Q waves and T wave inversions in leads I, aVL, and V3. These findings along with the conduction defects were all seen on previous ECGs. Review of prior records indicated that he had a left heart catheterization about 18 months prior to this admission. At that time was found to have nonobstructive coronary disease.

**Figure 2.**
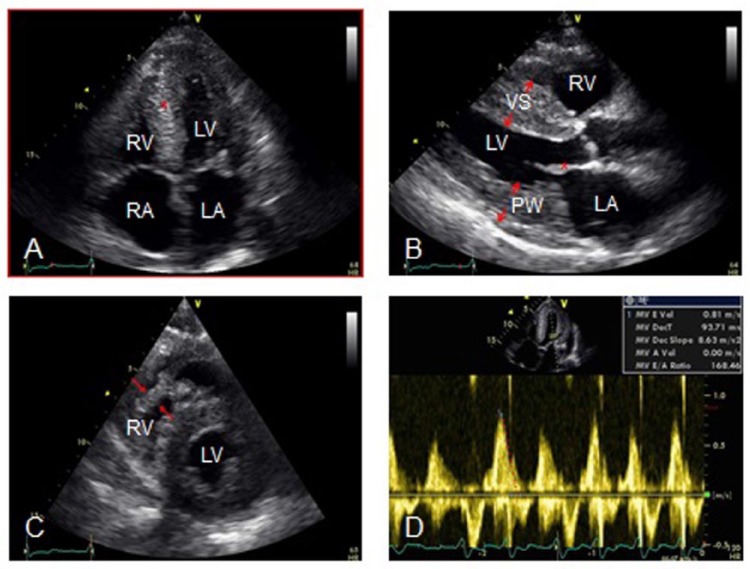
Two-dimensional echocardiogram. (A) Four-chamber view demonstrating bi-atrial enlargement (RA and LA) with speckled granular appearance of the myocardium (asterisk). (B) Parasternal long axis view demonstrating thickened ventricular septum (VS), posterior wall (PW), and thickened mitral valve leaflets (asterisk). (C) Short axis view demonstrating right ventricular hypertrophy (arrows). (D) Mitral valve inflow velocities demonstrating rapid deceleration time consistent with severe restrictive physiology. Abbreviations: RA, right atrium; LA, left atrium; RV, right ventricle; LV, left ventricle; VS, ventricular septum; PW, posterior wall.

His most recent echocardiogram (Figure 2), which was performed 2 weeks prior to this admission, showed a reduced ejection fraction (40%), biatrial enlargement (Figure 2A, RA and LA), and biventricular hypertrophy (Figure 2B, VS and PW; Figure 2C, arrows). There was also a granular or “speckled” appearance of the myocardium (Figure 2A, asterisk). The estimated peak right ventricular systolic pressure was 34 mm Hg based on an estimated right atrial pressure of 13 mm Hg, without primary valvular abnormalities. Mitral valve inflow Doppler and mitral annulus tissue Doppler (Figure 2D) were consistent with severe left ventricular diastolic dysfunction. The E/e ratio was >15, consistent with an elevated left ventricular filling pressure. There was hypokinesis to akinesis of the inferior and septal walls.

**Figure 3.**
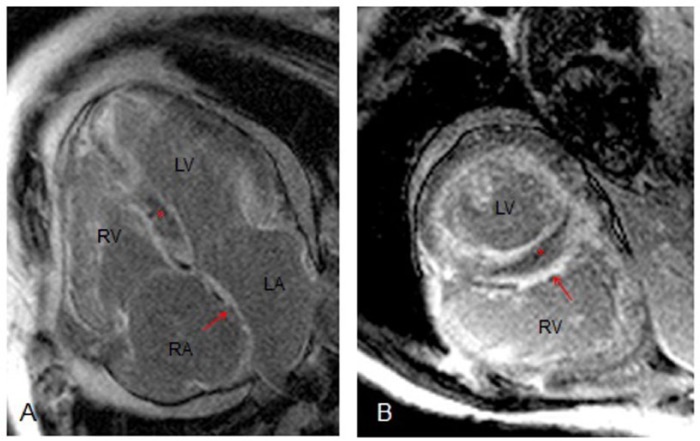
Cardiac MRI. (A) Four-chamber view demonstrating hyper enhancement (asterisk) typically found in cardiac amyloidosis and subendocardial scar (arrow). (B) Short axis view demonstrating hyperenhancement (asterisk) and subendocardial scar (arrow) pattern. Abbreviations: RA, right atrium; LA, left atrium; RV, right ventricle; LV, left ventricle.

The patient was monitored overnight and his symptoms resolved without intervention. His blood pressure remained within normal limits and he had normal urine output. The aminotransferases declined dramatically, to a level of 1070 IU/L within 48 hours. He was discharged home in baseline condition. One month later, he was seen in cardiology clinic during which time he had further evaluation with cardiac magnetic resonance imaging (MRI; Figure 3). This revealed diffuse myocardial thickening of both left and right ventricles with delayed hyperenhancement in a pattern consistent with amyloidosis (Figure 3A and B, asterisks). Both atria were dilated in size, with evidence of patchy subendocardial scar (Figure 3A and B, arrows). The patient declined endomyocardial biopsy and further invasive investigation or therapy.

## Discussion

The differential diagnosis of a rapid and dramatic rise in aminotransferases is limited and includes hepatocellular injury from toxins, viral infection, a passed gallstone, or ischemic hepatitis. Biliary tract disease, toxin-induced injury, and viral hepatitis were all ruled out, and the patient’s aminotransferases quickly improved with supportive care. In the context of the patient’s cardiac history, the clinical picture was diagnostic for ischemic hepatitis, and moreover shifted attention to the heart. With further investigation, the patient was found to have an infiltrative cardiomyopathy due to cardiac amyloidosis.

Ischemic hepatitis, also known as shock liver or hypoxic hepatitis, was described as early as 1901 in a series of autopsies that revealed homogenous hepatic necrosis distributed around the central veins.^1^ The clinical syndrome underlying this centrilobular necrosis involves a sudden and substantial rise in liver transaminases, presumably due to an acute hypoxic insult. It has been demonstrated that this process occurs in patients who already have an underlying disease that chronically compromises oxygen delivery to the liver.^2^ In the vast majority of patients, hypoxic hepatitis occurs in the context of underlying heart disease.^2,3^ In one study, all patients with clinically defined ischemic hepatitis were found to have primary organic heart disease.^4^ Importantly, a true shock state is not always present, which makes the name—“shock liver”—misleading (the preferred term is “hypoxic hepatitis” or “ischemic hepatitis”).^2,4^

The pathogenesis of ischemic hepatitis remains poorly elucidated. The liver receives approximately 25% of the body’s cardiac output, thus it is not surprising that disruption in cardiac function may ultimately affect the liver.^5^ Both passive congestion of the liver secondary to right heart failure and decreased cardiac output appear to be essential for hypoxic hepatitis to develop.^4,5^ Of note, passive congestion seems to play the more dominant role in compromise of oxygen delivery to the liver.^4-6^ It has been postulated that the liver needs to be “primed” by passive congestion in order to sustain hepatic necrosis caused by reduced cardiac output. Our patient had multiple cardiac abnormalities, but the most likely causative factors were his passive congestion due to his infiltrative myocardial disease and an additional episode of reduced arterial perfusion of the liver. The acute insult underlying the latter process was unclear at this time, but may have been due to his recent atrial fibrillation.

The prevalence of hypoxic hepatitis is likely underestimated for several reasons. First, the aminotransferase increase, although dramatic, typically resolves quickly and can be missed in the absence of frequent monitoring.^2,7,8^ Second, familiarity of clinicians with hypoxic hepatitis is often lacking. Finally, hypoxic hepatitis is a diagnosis of exclusion, and without expert consultation, the disease may be difficult to differentiate from other diseases.

The mortality rates of patients with hypoxic hepatitis is twice that of patients with liver enzyme elevations due to other causes.^9^ The absolute value of the liver enzymes themselves are not associated with prognosis, but the extent of hepatic injury, illustrated by INR elevation, has been shown to correlate with increased mortality. The duration of the elevated transaminases also has a significant impact on mortality.^10^

Infiltrative cardiac disease can be caused by a number of diseases including hemochromatosis, sarcoidosis, and amyloidosis. Regardless of the precise etiology, the cardiac pathophysiology is similar. Infiltrative diseases of the heart typically result in progressive diastolic dysfunction, which eventually leads to overt systolic dysfunction.^11^ Roughly 5% of initially unexplained cardiomyopathies are due to infiltrative heart disease, with amyloidosis accounting for the highest proportion.^12^

Amyloidosis is a condition in which amyloid fibrils are deposited into extracellular tissue. This disease can be systemic or involve just a single organ.^13^ With regard to the heart, amyloid fibril deposition can lead to biventricular wall thickening with impaired relaxation, local ischemia due to infiltration of myocardial vessels, and conduction abnormalities and arrhythmias.^14^ The initial finding of wall thickening is often misdiagnosed as left ventricular hypertrophy due to hypertension. Endomyocardial biopsy is the gold standard for diagnosis of cardiac amyloid; however, newer techniques such as cardiac MRI are nearly as accurate. A distinct pattern of late gadolinium enhancement of the subendocardium on cardiac MRI, as we present in our patient, can be used to noninvasively diagnose cardiac amyloidosis with a sensitivity of 80% and specificity of 94%.^15^

## Conclusion

In summary, an abrupt elevation in aminotransferases without a clear cause often points toward ischemic hepatitis. This case highlights the point that cardiovascular dysfunction is often overlooked in patients with ischemic hepatitis, and infiltrative cardiac processes should be considered in patients without obvious primary organic heart disease.
